# Thinking more wisely: using the Socratic method to develop critical thinking skills amongst healthcare students

**DOI:** 10.1186/s12909-023-04134-2

**Published:** 2023-03-20

**Authors:** Yueh-Ren Ho, Bao-Yu Chen, Chien-Ming Li

**Affiliations:** 1 Department of Biochemistry and Molecular Biology, College of Medicine, National Cheng Kung University, University Road No.1, East District 701, Tainan City, Taiwan (R.O.C.); 2 School of Medicine, College of Medicine, National Cheng Kung University, University Road No.1, East District 701, Tainan City, Taiwan (R.O.C.); 3 Institute of Clinical Medicine, College of Medicine, National Cheng Kung University, University Road No.1, East District 701, Tainan City, Taiwan (R.O.C.); 4 Division of Infectious Diseases, Department of Internal Medicine, Chi Mei Medical Center, Zhonghua Raod No.901, Yongkang District 710, Tainan City, Taiwan (R.O.C.)

**Keywords:** Biochemistry experiment, Critical thinking, Socratic method, Medical education, Metacognition

## Abstract

**Background:**

In medicine, critical thinking is required for managing and tolerating medical uncertainty, as well as solving professional problems and treating diseases. However, the core of Confucianism, teacher-centered and exam-oriented settings in middle and high school education may pose challenges to developing critical thinking in Han Chinese or Taiwanese students. Students may be adversely affected by these pedagogies since student-centered settings were more effective in stimulating their critical and reflective thinking, as well as a sense of responsibility, in the ever-changing world. Therefore, guiding students with less stable foundations of critical thinking might require a different approach. A review article highlighted the potential utility of the Socratic method as a tool for teaching critical thinking in the healthcare field. The method involves posing a series of questions to students. More importantly, medical students and residents in clinical teaching are familiar with the method. Almost all healthcare students must complete a biochemistry laboratory course as part of their basic science training. Thus, we aimed to train students to develop critical thinking in the biochemistry laboratory course by using learning sheets and teacher guidance based on the Socratic method and questioning.

**Method:**

We recruited second-year students from a medical school, of whom 32 had medical science and biotechnology majors (MSB), 27 had pharmaceutical science majors (PS), and 85 were medical undergraduate (MU) students. An exercise in critical thinking was conducted during a biochemistry laboratory course, which consisted of five different biochemical experiments, along with learning sheets that contained three or four critical thinking questions. Then, the teacher evaluated the students’ ability to think critically based on nine intellectual dimensions (clarity, accuracy, precision, relevance, depth, breadth, logic, fairness, and significance) based on the universal intellectual standards developed by Prof. Linda Elder and Richard Paul. In the following analysis, regression models and multivariate analysis were used to determine how students improved over time, and trajectory analysis were carried out in order to observe the trends in students’ critical thinking skills construction.

**Results:**

Clarity and logic dimensions were identified as the key elements to facilitate the development of critical thinking skills through learning sheets and teacher guidance in students across all three different healthcare majors. The results showed that metacognitive monitoring via Socratic questioning learning sheets have demonstrated potential encourage students to develop critical thinking skills in all dimensions. Another unique contribution of current study was present the heterogeneous learning patterns and progress trajectories of clarity and logic dimensions within classes.

**Conclusion:**

Using the Socratic learning model could effectively develop students’ critical thinking skills so they can more effectively care for their patients.

**Supplementary Information:**

The online version contains supplementary material available at 10.1186/s12909-023-04134-2.

## Introduction

Emerging trends in information technology requires that the new generation of medical students become critical thinkers [1]. The General Medical Council (GMC) of the United Kingdom encourages teachers to facilitate the acquisition of critical thinking skills by students in the medical and health professions [2]. Decades of research have proven that critical thinkers can present dispositions like flexibility, persistence, and willingness when faced with a range of tasks; they display meta-cognitive monitoring and a willingness to self-correct to seek long-term consensus[3]. Although, critical thinking is constructed from childhood in most Western countries and are valued by higher education as a necessary skill for coping with society [4]. However, critical thinking constructing and teaching has attracted little attention in Eastern education systems until recently [5, 6].

Aside from the development of critical thinking skills is a key component of educational systems, recent educational philosophy also emphasizes both thinking processes as well as metacognitive integration skills [7]. Metacognitive monitoring includes making ease-of-learning judgments (i.e., processing fluency and beliefs), judgments of learning, feeling-of-knowing judgments (i.e., assessing the familiarity of the cue and the question itself or the domain of the question), and having confidence in the retrieved answers [8, 9]. It is an adaptive skill of personal insight that health-profession students need to succeed in the rapidly changing and challenging healthcare industry [2, 10]. Despite this, higher education curriculum does not emphasize on teaching these skills [7]. Additionally, any attempts to change the standards in higher education are generally met with resistance and challenges since they are require to encourage teachers to create new curriculum and change the current teaching content by researchers in current study who have more than 40 years’ teaching experience observaions. Healthcare curriculum, in general, remains conservative; Taiwan is not an exception.

Critical thinking is a fundamental component of innovative thinking and has thus become the fundamental skill for cultivating innovative talents in Western education [11]. Western scholars have asserted that teaching critical thinking should start at an early age and that its foundations should be laid in elementary and secondary schools. There are many ways to define critical thinking. A leading educational expert, Prof. Dewey, defined critical thinking as inclusive of reflective thinking and argued that the thinking process should also be taken as one of the objectives of education [12]. There are a few general dispositions that an ideal critical thinker would present according to Prof. Ennis’ observation of the constitutive abilities, such as (1) provide a clear statement of the conclusion or question; (2) provide clear reasons and be specific about their relationships with each other; (3) try to be well informed; (4) always seek and use credible sources, observations and mention them frequently; (5) consider the entire situation; (6) be mindful of the context’s primary concern; (7) be aware of alternative options; (8) be open-minded toward other points of view and refrain from making a judgment when there are insufficient evidence and reasons; (9) be willing to change your position when sufficient evidence and reasons support it; (10) seek as much precision as the nature of the subject admits; (11) whenever possible, seek the truth, and more broadly, strive to “get it right”; and (12) utilize their critical thinking abilities and dispositions [13–16]. In the eyes of Profs. Dewey and Ennis, critical thinking is a process of careful thought and reflection before a decision is made [17].

Nevertheless, the measurement or evaluation of critical thinking skills and abilities does not seem easy. Based on another perspective on critical thinking, intellectual standards are evolving [18]. According to Profs. Elder and Paul, critical thinking is the ability to use the most appropriate reasoning in any situation [18]. To evaluate these abilities, they established nine dimensions of critical thinking to represent different aspects of critical thinking: clarity, accuracy, precision, relevance, depth, breadth, logic, significance, and fairness [18]. As Profs. Elder and Paul concluded, those who possess discipline and critical thinking skills would make use of intellectual standards every day; thus, people should target these standards when they ask questions during the thinking process [18, 19]. As a result of teachers’ regular introduction of the tools of critical thinking in their classrooms, the Socratic questioning and discussions become more productive and disciplined, thereby enabling students to realize the significance of questioning during the learning process [20–22].

According to a review article, teaching critical thinking to healthcare students (primarily medical and pharmacy students) through Socratic methods is more effective in developing critical thinking for a number of reasons [23]. In particular, Socratic questioning provides students with the opportunity to justify their own preconceived beliefs and thoughts after a series of specific, targeted inquiries [24]. Using Socratic questioning can also assist healthcare students, interns, or residents in thinking critically by understanding the “deep structure” of the question, i.e., deconstructing the question and understanding its true meaning [23]. The effectiveness of Socratic questioning lies in ascertaining the current knowledge of the students [25] and establishing a foundation for teaching at their level [26]. The teacher can accomplish this probing by asking progressively more challenging questions until the limits of the students’ knowledge are discovered [25, 27, 28], as well as by allowing students to express their existing knowledge, which in turn will allow them to synthesize new knowledge [26], and the dialogue represents the Socratic method [29]. Alternatively, a critical thinker is more likely to engage in certain established metacognitive strategies under the Socratic paradigm and/or channel the intellectual dimensions of critical thinking [17].

Unfortunately, Han Chinese students have struggled with learning critical thinking, which is thought to be part of their characterological profile [30]. This struggle has been faced by students studying abroad [11] and in students enrolled in the Han Chinese education system, which mainly cultivates Confucianism [31]. There are at least two types of problems with developing critical thinking in Han Chinese or Taiwanese education. The first involves the core of Confucianism, where foreign teachers have tried to promote critical thinking in elementary and high schools but sensed ethical concerns from the students who refused to participate. This is likely because if they chose to participate, they would have felt obligated to express disagreement and negative feelings to the instructor. The Han Chinese culture values harmony and “not losing face,” emphasizing a holistic perspective and collective good. Thus, students would feel uncomfortable because disagreeing with someone’s opinion in public is consciously or often avoided [30]. Therefore, encouraging the student to participate in healthy discussions and respectfully challenge their teachers is the starting point for promoting critical thinking in students enrolled in the Han Chinese educational system.

Second, in the Western education approach, learners take an active role in and are responsible for their learning process. On the contrary, the Han Chinese and Taiwan education systems are teacher-centered and exam-oriented; students are expected to follow their teachers’ instructions and perform well in class. More importantly, the textbook or teacher-centered framework lacks half of Ennis’s twelve constitutive abilities for critical thinking [13–15], such as judging the credibility of a source, observing and judging observation reports, drawing explanatory conclusions (including hypotheses), making and judging value judgments, and attributing unstated assumptions. As a result, Han Chinese students may find it difficult to develop critical thinking skills and present key traits and dispositions that are indicative of an ideal critical thinker. Hence, guiding and evaluating critical thinking in students might not be implemented through the same approach in Eastern educational circumstances as in the West. By understanding the difficulties that Han Chinese students face in developing critical thinking, the current study aims to design a set of critical thinking models that are suitable for Han Chinese students as a starting point for reform teaching.

## Research questions, hypotheses and objectives

**Fig. 1:**
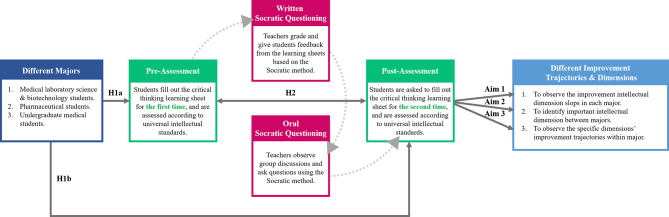
Socratic method framework and structure of the research hypotheses behind the biochemistry laboratory course

Research has shown that the laboratory class is not just limited to a step-wise approach to experimentation. It also allows students to develop their critical thinking skills by repeatedly engaging a simple learning framework [32]. To explore this further, the current study’s primary purpose is to use Socratic questioning in a biochemistry laboratory course with specifically designed learning sheets and feedback from teacher to guide students to improve their critical thinking skills. The learning sheets were evaluated following the universal intellectual standards for critical thinking developed by Prof. Elder and Paul [19, 33]. For this study, we hypothesized that students with different healthcare majors might present different improvement trajectories in their intellectual dimensions according to the years of teaching observations in the three healthcare majors. Based on the research and rationale described above, the intervention effect of Socratic questioning in a biochemistry laboratory course was hypothesized as follows (see Fig. 1):

1. Pre-intervention critical thinking abilities are different amongst students of different healthcare majors, especially in each intellectual dimension (H1a). Post-intervention critical thinking abilities would develop in students from each healthcare major after using the Socratic method (H1b).
2. Critical thinking abilities differs significantly between pre- and post-assessments of the intellectual dimensions of students with the three different healthcare majors (H2).
3. After clarifying the relation of Socratic method interventions in the class, we aim to scrutinize the trajectories of students between majors further to understand the learning style in class (Aim 1). Furthermore, we also aim to identify the key intellectual dimensions that could lead to an overall improvement in the critical thinking of students in each major (Aim 2). Additionally, we observed improvement trajectories of specific intellectual dimensions within major (Aim 3).

## Literature review

### Critical thinking engagement in the Eastern and Western medical education

Over the last decade, medical education has been undergoing a variety of approaches for effectiveness teaching and transformation [34]. Many paradigms of active teaching/learning methodologies have been adopted in both Eastern and Western medical education systems, some of which are used partially (actual or conceptual similar) Socratic questioning to challenge students’ critical thinking. In this regard, the primary philosophy of case-based learning (CBL) established in the 1920s by Harvard Medical School is to guide students to apply their acquired knowledge base via critical thinking to make clinical decisions to solve the problems that they may encounter in the healthcare environment [35]. A meta-analysis study of China’s dental education reported that the CBL was a practical pedagogical method across the Chinese dental education system [36]. The results showed that the CBL method significantly increased knowledge scores, skill scores, comprehensive ability scores, and teaching satisfaction compared with the traditional lecture-based learning (LBL) mode in 2,356 dental students. Hence, there is an urgent need to change the traditional didactic lecture or teacher-centered classroom setting in which students are passive listeners instead of active participants.

Healthcare professionals are also required to solve complex problems and efficiently integrate didactic preclinical knowledge into actual clinical application in patient care [35]. On the other hand, the design thinking process may enhance both creativity and innovation so that healthcare professionals can respond to clinical problems effectively [37, 38]. Problem-based learning (PBL) is a pedagogical approach widely accepted in medical education. It promotes active learning and results in better outcomes [39–41]. PBL focuses on active lifelong learning by triggering problems, directing student focus, and facilitating tutor involvement [39, 42–44]. However, it is noteworthy that some hybrid PBL models have become less effective over time, as well as less aligned with the intended philosophy of student-centered learning [45]. Another alternative blended learning approach of PBL is team-based learning (TBL), which allows medical educators to provide students with pre-class work, in-class initial tests with immediate feedback, and real clinical problem-solving activities [46]. In the year-one studies of the Sydney Medical Program, a greater level of engagement in learning, a deeper understanding of concepts, and a sense of responsibility were shown among the medical students working in a TBL setting than among those in a PBL setting [47, 48].

Medical educators face another significant challenge with the millennial generation, which has ubiquitous information technology access throughout its education. Thus, it is extremely important to improve students’ motivation to learn through hands-on instruction or teacher–student interaction and then stimulate students’ thinking and learning. In recent years, gamification has been successfully integrated into medical and scientific endeavors, enhancing motivation, participation, and time commitment across a variety of settings [49–51]. Another healthcare curriculum reform to stimulate active learning is flipped classroom (FC), which assigns learners didactic material, creating opportunities of longitudinal and interprofessional learning experiences for students during class participation [52] to encourage extracurricular learning, such as critical thinking. As part of the FC model, medical educators also develop formative and diagnostic assessments to identify learning gaps. According to these teaching modules, encouraging students to participate, emphasizing their learning, and observing their development trajectory are the core ideas in recent educational designs [53].

Although most of above-mentioned studies have been performed in the Eastern and Western education systems, however, without mentioning the differences between cultures and learning styles. Most importantly, the cultivation and foundations of critical thinking neglect the fact that Eastern and Western education systems emerged from very different learning and thinking patterns. Moreover, clinical reasoning and decision achievements depend on established critical thinking skills, therefore, it becomes more important to construct critical thinking early and comprehensively [54]. While Han Chinese students are not familiar with the core of critical thinking, the most effective approach to teaching critical thinking is still a highly debated topic in medical schools. Taken Taiwan medical education as an example, most clinical courses focuses on professional skills, problem solving, and disease treatment rather than construct critical mindset and metacognitive skills. Education strategies often emphasize the outcome while neglecting the process. Nevertheless, medical educators should also emphasize the process of forming students’ critical thinking when instructing and guiding them in this regard. Consequently, using metacognitive monitoring to enhance critical thinking in healthcare education would be appropriate, especially for Han Chinese systems with a Confucianist outlook. Thus, critical thinking via metacognitive monitoring is important in healthcare education, especially in Han Chinese systems with a Confucianist background.

### Proficiency in the art of socratic questioning to enhance students’ critical thinking

Socratic questioning is a disciplined method of engaging in content-driven discourse that can be applied for various purposes: analyzing concepts, finding out the truth, examining assumptions, uncovering assumptions, understanding concepts, distinguishing knowledge from ignorance, and following the logical implications of thought. The scholars who established the intellectual standards of critical thinking have consistently indicated that “The key to distinguishing it from other types of questioning is that the Socratic questioning is systemic, disciplined, and deep and usually focus on foundational concepts, principles, theories, issues, or problems [20–22].” In short, the Socratic method is a questioning method that stimulates personal understanding. More importantly, the core principle of learning from the unknown fits best within healthcare environments.

Numerous studies have consistently urged teachers to develop Socratic dialogue in their classrooms, regardless of their learning stages and situations [55–57]. Using enhancement exercises in an elementary school, a study introduced a Socratic questioning strategy to provide guidance and hints to students so that they could think more deeply about an issue or problem before sharing their thoughts [55]. The lecturer of a speech course in higher education demonstrated how Socratic questioning could help students learn when confronted with a series of questions [56]. The process improves students’ ability to ask and answer questions and helps them overcome some obstacles related to their lack of self-confidence. In the book *Socratic circles: Fostering critical and creative thinking in middle and high school*, Dr. Matt Copeland stated that, in middle and high schools, teachers must facilitate discussions by asking questions [58]. Furthermore, this method could be applied not only to elementary school, middle school, high school but also to higher education classes [59]. During the Covid-19 pandemic, synchronous discussions in online learning demonstrated that the Socratic questioning strategy successfully improves students’ critical thinking skills [57].

The incorporation of Socratic questioning in healthcare education curriculum is under development, including for general medical education [60], medical [61], pharmacy [54, 62], and nursing students [63]. A review article of revisiting the Socratic method as a tool for teaching critical thinking in healthcare professions revels few advantages of Socratic questioning [23]. Three type of Socratic questions were mention and could commonly used in different clinical situations [23], such as procedure question would use in those with correct answers (e.g., *Which of the following medications has antithrombotic function?*); preference question can apply in those with no correct answers (e.g., *What type of consultation is most suitable for this patient?*); judgment question would be the most challenge critical thinking within a Socratic paradigm by integrating different domain knowledge and skills (e.g., *Does this patient require antibiotic treatment?*). It is necessary to apply and analyze information in a logical manner as well as self-regulate and use critical thinking in order to achieve the best outcome for patients. For medical doctors, pharmacists or clinical laboratory technicians to provide high quality health care across all disciplines, critical thinking is inherently required.

In medical school, the emphasis is laid on training learners in meta-capabilities, such as self-driven pattern recognition, ideally as part of an apprenticeship under the supervision of an expert diagnostician [61]. An in-depth study of the current trends in developing critical thinking amongst medical students demonstrated the use of dialogue for proper questioning and how it directs the learner’s thinking [64]. Moreover, another study confirmed that critical thinking occurs only when students are motivated and challenged to engage in higher-level thought processes [65]. In the pharmacy classroom, educators can play a significant role in influencing their students’ mindsets.  Growth mindsets can be cultivated through the creation of an environment that encourages it. [62]. The Socratic questioning method can facilitate critical thinking in nursing education. One study showed that problem solving using critical thinking skills can be facilitated in both educational and practice settings by using Socratic inquiry [63].

The Socratic method has been adapted in different ways to different domains, but it has become closely associated with many areas, such as basic scientific thinking training, legal dialectical guidance, and clinical teaching. Some adaptations are helpful, some are not. The adaptations can be looked at through reasoning-focused lenses with varying degrees of magnification —a high-magnification adaptation rigorously and precisely tracks or guides the path of reasoning. Thus, how to use the Socratic method to direct students onto the path of critical thinking with appropriate guidance, but not revealing answers becomes an art that tests instructors’ teaching experience and proficiency in questioning.

### Critical thinking and reflection exercises in the laboratory course

Medical schools have increasingly encouraged students to become life-long, self-directed learners because of the continual changes in the evidence-based healthcare environment. Science is often applied in everyday life, including translating knowledge from scholarly fields [66]. However, there is a vast gap between what is taught in medical schools and what is actually required in practice has increasingly widened in this information era. The majority of healthcare professionals are not considered to be real scientists. [2]. Nevertheless, they need to know how to apply scientific knowledge to their practice. Therefore, a science curriculum in medical school, such as a biochemistry laboratory course, should provide an opportunity to learn scientific methods and conceptual frameworks. It should also promote critical reasoning, providing healthcare students with problem-solving skills.

Medical educators need to accept that critical thinking is important for healthcare students and know how to teach it effectively [67]. Medical educators are now faced with a dilemma: should they develop a new course or adapt old course to develop critical thinking skills?  An effective learning model should promote and stimulate students’ development of such skills [67]. One of the most common compulsory courses for healthcare students is the biochemistry laboratory course [68, 69]. These courses are specifically designed to introduce students to prescribed experiments, requiring them to complete stepwise protocols by themselves [68, 70]. The students are expected to understand the concepts behind the methods, procedures, and assays. However, this type of curriculum construction often fails to provide students with adequate opportunities to monitor their critical thinking and thus reduces the chances of developing problem-solving skills [70]. In order to provide students with more opportunities to think critically, previous studies have also adapted laboratory, basic science, and science fusion courses to help students develop critical thinking skills [67, 68, 71–73].

Several studies have demonstrated that students need critical thinking skills to interpret data and formulate arguments. Thus, science education, particularly in the laboratory setting, is designed to teach quantitative critical thinking (i.e. interpretation and critical evaluation of statistical reports), but the evidence has suggested that this is seldom, if ever, achieved [74–79]. By providing multiple opportunities for students to participate in critical thinking in the physics laboratory classes at Stanford University, scholars engaged the students to improve the experiment and modify the model repeatedly [32]. Additionally, a simple learning framework using decision-making cycles and demonstrating experts’ critical thinking significantly improved students’ critical thinking. We thus argue that students should engage in critical thinking exercises with repeated comparisons, decisions, and teacher guidance that are meant to construct their critical thinking in each of their disciplines.

## Methods

### Participants

This research was conducted during the 2017–2018 academic year. The participants were second-year students in the College of Medicine at the National Cheng Kung University (NCKU) of Taiwan. A total of 144 students participated in this study, of whom 32 had medical science and biotechnology majors (hereafter, MSB), 27 had pharmaceutical science majors (hereafter, PS), and 85 were medical undergraduate (hereafter, MU) students. The biochemistry laboratory course was compulsory for these three majors.

### Procedure

For each biochemistry laboratory class, the teacher assembled five to six groups of four to five students each. The course contained five different biochemical experiments: (1) Plasmid DNA (deoxyribonucleic acid) extraction and purification; (2) restriction enzyme digestion and electrophoresis of plasmid DNA; (3) polymerase chain reaction (PCR) amplification of plasmid DNA; (4) recombinant protein expression in *Escherichia coli*; and (5) quantification of recombinant protein. The experimental learning sheets included three or four critical thinking questions (Table S1), encouraging students to explore experimental principles and alternative explanations further. To facilitate discussion, students were organized into small groups of four to five students seated around a single table, discussing and answering the questions. At this time, the students would pen down their first answers to the critical thinking questions, and the teacher would grade them based on the universal intellectual standards (learning sheets, first evaluation).

Furthermore, according to the students’ answers, the teacher offered a response by asking more questions according to the Socratic method to encourage students to think deeper rather than provide the correct answers. At the following week’s class, the teacher returned the learning sheet and supervised the ongoing activity, clarifying any questions raised by students and encouraging them to re-discuss and re-answer the critical thinking questions according to the teacher’s suggestions. The objective was to create a highly interactive environment to engage students in learning the relevant principles of each laboratory, including troubleshooting experiments and formulating critical concepts and skills. After the discussion, the teacher reexamined the students’ responses and assessed them based on the universal intellectual standards for subsequent grading (learning sheets, second evaluation).

The biochemistry laboratory courses and the Socratic method in current study are performed and taught by a senior biochemistry teacher (PhD in Institute of Basic Medical Science, NCKU) who has 40 years teaching experience. The teacher has long focused on teaching critical thinking skills to students, and also offers four senior clinical case related courses by practicing the Socratic method, such as clinical concept, critical thinking in medicine, clinical reasoning and special topics in clinical reasoning with more than 20 years of experience. Therefore, in the course, teacher will often ask a series of questions for students to think about the relevance of biochemical science and clinical practice.

### Assessment development

The research team designed the learning sheets to guide discussion on the key issues concerning five biochemical experiments. The learning sheets were assessed according to the universal intellectual standards for critical thinking [33]. However, the assessment was adapted to include nine intellectual dimensions to assess student reasoning [19, 33]: clarity, accuracy, precision, relevance, depth, breadth, logic, fairness, and significance (Table S2). Each dimension was evaluated using a binary score (0 = does not present the skill; 1 = presents the skill) for each question in the learning sheets for both the first and second evaluations. The students received the teacher’s guidance following the first evaluation, providing them with the opportunity to reconsider their reasoning and revise their answers. Our goal was to improve our students’ learning by stimulating the teaching process; at the same time, we were committed to allowing students to speak freely so that we could more effectively facilitate prospective discussions. Thus, the critical thinking scoring system based on nine intellectual dimensions was only for the purpose of the research, without consequences on students’ study progress. In this regard, students were not able to know their intellectual scores. As a result, their course grades were not determined by the learning sheets; rather, they were determined by the general operation, experiment report, and the learning attitude demonstrated during the experiments.

### Statistical analysis

#### Descriptive statistics and variable tests

We calculated the differences between the performance means for the first and second evaluations using paired *t*-tests. The mean differences between the students from the three majors were analyzed using a one-way analysis of variance (ANOVA). For the improvement slope for each universal intellectual dimension, we used the second evaluation scores of each experiment as the point with which to construct a quadratic equation curve in one variable (dimension) and then access the slope to represent the students’ improvement. The higher the slope score, the greater the students’ progress on that dimension.

### Multivariate analysis

We used traditional analytical methods to observe and analyze the students’ improvement in the five experiments. Data from the second evaluation scores of each experiment served as the multi-time point measurement data. The Cox regression model for multivariate analysis was used to investigate the effect of several variables upon the time during which a specified outcome happened [80]. For each dimension, the model’s outcome determined that a student’s improvement slope was defined as minor progress if it was lower than the improvement slopes of their peers in the same major overall. However, if the student’s improvement slope was higher than the overall progress intercept of their peers, then it was defined as greater progress. The Cox regression models’ outcomes for each dimension were divided into two groups: minor and more progress. For this model’s outcome, (1) we calculated all dimensions’ slopes mean from each major (MSB: 0.369; PS: 0.405; MU: 0.401); (2) then compared the mean slope of the individual students with the mean slope of major; (3) if the student’s individual improvement slope was lower than mean slope of major, then defined as minor progress; if the student’s individual improvement slope was higher than mean slope of major, then defined as greater progress. From the analysis at this point, we understood that teacher could help students from different majors develop the different dimensions of critical thinking with the use of Socratic methods and simple repeated thinking framework practice. Additionally, we wanted to represent the improvement of intellectual dimensions between the students of different majors and their heterogeneity in critical thinking.

#### Dimension identification and comparison

To understand which intellectual dimensions were most representative of student improvement across majors, the analysis was divided into three sections: (1) to identify the progress percentage of all nine intellectual dimensions; (2) to identify the progress percentage of statistically significant intellectual dimensions; (3) to compare the differences among all nine dimensions, the significant dimensions, and the reciprocal dimensions. This analysis offered a better understanding of what dimensions represented the overall improvement of students’ critical thinking. Our first step was to calculate the percentage of improvement for each experiment by determining the results of the first and second evaluations for each intellectual dimension. Second, we took average percentage of improvements for each dimension. Finally, we used Student’s *t*-test to compare the differences among the average of all nine dimensions, the significant dimensions, and the reciprocal dimensions.

### Trajectory analysis

In this study, we also hypothesized that each student’s learning and progress trajectories were heterogeneous across different majors. Depending on the major, there may also be differences between students in the same class. To focus our observations on the students’ use of the clarity and logic dimensions, we used a trajectory-tracking analysis [81, 82] and categorized the students into two groups based on the participants’ improvement levels within the same major.

## Results

### Descriptive data

We recruited 144 second-year students from three majors in the College of Medicine, among which 32 were MSB, 27 were PS, and 85 were MU students. All participants’ first and second evaluations were compared in all five biochemistry experiments. The statistically significant between-group differences in the mean initial evaluation results for each dimension are presented in Table 1.

**Table 1:** Description of the participants and their performance in the evaluations of their learning sheets (N = 144)

	Medical laboratory science & biotechnology students^†^	Pharmaceutical students^†^	Undergraduate medical students^†^			
**Characteristics**	***N (%)***	***N (%)***	***N (%)***	***ANOVA*** ^**#**^		
	32 (22)	27 (19)	85 (59)			
	***Mean (SD)***	***Mean (SD)***	***Mean (SD)***	***df***	***F***	***p***
**Learning sheets** ^**‡**^	***1*** ^***st***^ ***/ 2*** ^***nd***^ ***evaluation*** ^*^	***1*** ^***st***^ ***/ 2*** ^***nd***^ ***evaluation*** ^*^	***1*** ^***st***^ ***/ 2*** ^***nd***^ ***evaluation*** ^*^			
Total score	13.09 (1.35) / 23.30 (1.12)^*^	15.28 (5.36)^a^ / 25.13 (5.89)^* a^	15.82 (6.56)^c^ / 24.72 (6.98)^* c^	2	3.05	0.0478
Clarity	2.57 (1.20) / 3.23 (0.72)^*^	3.02 (0.72)^a, b^ / 3.51 (0.53)^* a, b^	2.79 (1.00)^c^ / 3.34 (0.69)^*^	2	0.30	0.0019
Accuracy	1.65 (1.35) / 2.77 (1.12)^*^	1.93 (1.15) / 2.87 (0.92)^*^	2.02 (1.20)^c^ / 2.85 (0.85)^*^	2	0.62	0.5406
Precision	2.17 (0.96) / 3.10 (0.82)^*^	2.64 (0.89)^a, b^ / 3.23 (0.75)^*^	2.43 (1.04)^c^ / 3.05 (0.77)^*^	2	1.10	0.3647
Relevance	2.31 (1.15) / 3.01 (0.91)^*^	2.35 (0.92) / 3.16 (0.72)^* b^	2.31 (1.04) / 2.97 (0.87)^*^	2	2.66	0.0707
Depth	0.85 (1.01) / 2.29 (1.17)^*^	1.07 (0.75) / 2.57 (0.96)^* a^	1.16 (0.92)^c^ / 2.59 (1.05)^* c^	2	4.67	0.0097
Breadth	0.57 (0.72) / 1.61 (1.06)^*^	0.67 (0.68) / 1.68 (1.09)^* b^	0.83 (0.77)^b, c^ / 2.04 (1.14)^* c^	2	11.28	< 0.0001
Logic	1.46 (1.28) / 2.74 (1.04)^*^	1.68 (1.23) / 3.03 (0.88)^* a,b^	2.01 (1.24)^b, c^ / 2.84 (0.98)^*^	2	3.31	0.0371
Significance	0.73 (0.85) / 2.15 (1.10)^*^	1.02 (0.80)^a^ / 2.52 (0.93)^* a^	1.11 (0.87)^c^ / 2.42 (1.00)^* c^	2	5.64	0.0037
Fairness	0.77 (1.88) / 2.40 (1.16)^*^	0.95 (0.79) / 2.56 (0.92)^*^	1.16 (0.87)^b,c^ / 2.53 (0.97)^*^	2	1.18	0.3085

^**†**^Students were divided into groups of 4–5 participants to complete the exercises. However, the learning sheets scores were filed individually. ^**#**^The difference between groups in their performance in the second evaluation was calculated using analysis of variance (ANOVA).

^**‡**^The subscales in the learning sheets are scored on a scale of 1–4 for each dimension.

^*^ The difference in performance between the first and second evaluations was compared using paired *t*-tests, *p* < 0.05.

^a^ Medical laboratory science and biotechnology vs. pharmaceutical students, *p* < 0.05.

^b^ Pharmaceutical vs. undergraduate medical students, *p* < 0.05.

^c^ Medical laboratory science and biotechnology vs. undergraduate medical students, *p* < 0.05.

### Overall improvement from the initial to second evaluations throughout the five experiments (H1, H2, and Aim 1)

Table 1 presents the mean results of the first and second evaluations; the five experiments exhibited statistically significant differences (*p* < 0.05) across all study groups and dimensions. More detailed analyses revealed significant differences in performance in the second evaluation between the groups after all five biochemistry experiments in the clarity (*p* = 0.0019), depth (*p* = 0.0097), breadth (*p* < 0.0001), logic (*p* = 0.0371), and significance (*p* = 0.0037) dimensions. However, for some of the dimensions (clarity, accuracy, precision, logic, and fairness), the initial evaluation results differ significantly between the MU and the MSB students, but this was not the case for the secondary evaluation results. The MSB students exhibited the best progress (2nd mean score minus 1st mean score) in the clarity dimension across all experiments. The PS students exhibited the best performance in the logic dimension (*p* < 0.05) in the second evaluation after the five experiments.

The results of the MSB students improved steeply in most dimensions in the five experiments, especially depth (slope: 0.472), logic (0.455), and clarity (0.410) (Table 2). Time had a stronger effect on several of the dimensions in the multivariate analysis, specifically clarity (*p* = 0.0012), relevance (*p* = 0.0007), and logic (*p* < 0.0001). By contrast, the PS students showed a significant overall improvement in the clarity (slope: 0.212, *p* < 0.0001), accuracy (0.539, *p* = 0.0063), precision (0.381, *p* = 0.0085), relevance (0.216, *p* < 0.0001), breadth (0.426, *p* = 0.0045), and logic (0.515, *p* = 0.0027) dimensions over the observation period (Table 3). Finally, the MU students showed a significant overall improvement in six dimensions: clarity (slope: 0.277, *p* < 0.0001), accuracy (0.520, *p* = 0.0003), depth (0.459, *p* = 0.0092), breadth (0.356, *p* = 0.0100), logic (0.544, *p* = 0.0190), and significance (0.327, *p* = 0.0225) (Table 4).

**Table 2:** Medical laboratory science and biotechnology students’ overall improvement throughout the five experiments (N = 32)

	*Learning sheets 1* ^*st*^ */ 2* ^*nd*^ *evaluation*						*Overall improvement*	
	**Mean (SD)**					**Slope** ^**†**^	**Multivariate regression**	
**Experiment**	**1**	**2**	**3**	**4**	**5**		**95% CI**	***p***
Dimension								
Clarity	1.64(1.07)/3.07(0.78)^*^	1.94(1.13)/2.66(0.48)^*^	3.06(0.88)/3.91(0.71)^*^	3.81(0.59)/2.91(0.30)^*^	2.44(0.84)/2.91(0.30)^*^	0.410	0.45–1.07	0.0012
Accuracy	0.47(0.79)/1.77(1.25)^*^	1.03(1.12)/2.25(1.08)^*^	2.03(1.03)/3.78(0.72)^*^	3.06(1.32)/2.53(0.42)^*^	1.75(0.80)/2.07(0.51)^*^	0.348	1.35–3.43	0.0995
Precision	1.21(1.01)/2.53(0.97)^*^	2.03(0.86)/2.66(0.70)^*^	2.25(0.62)/3.91(0.83)^*^	2.91(0.78)/3.90(0.30)^*^	2.53(0.51)/3.00(0.08)^*^	0.296	0.30–0.64	0.0659
Relevance	1.85(1.23)/2.47(1.20)^*^	2.03(0.69)/2.78(0.42)^*^	2.19(1.00)/ 3.91(0.70)^*^	3.38(1.26)/3.91(0.30)^*^	2.13(0.79)/2.50(0.72)^*^	0.172	0.69–1.93	0.0007
Depth	0.26(0.75)/1.77(1.25)^*^	0.41(0.71)/1.69(1.09)^*^	0.81(0.64)/3.25(1.29)^*^	1.88(1.34)/2.19(0.62)^*^	0.94(0.56)/2.37(0.78)^*^	0.472	1.28–2.55	0.5733
Breadth	0.32(0.77)/1.37(1.27)^*^	0.46(0.72)/1.41(0.80)^*^	0.59(0.72)/2.13(1.07)^*^	0.94(0.76)/1.50(1.04)^*^	0.56(0.50)/1.50(0.98)^*^	0.360	0.45–1.03	0.6305
Logic	0.26(0.75)/1.77(1.25)^*^	0.75(0.95)/2.25(0.95)^*^	1.47(0.67)/3.66(0.78)^*^	2.94(1.13)/2.88(0.48)^*^	1.94(0.80)/2.88(0.34)^*^	0.455	0.29–0.64	< 0.0001
Significance	0.26(0.75)/1.77(1.25)^*^	0.53(0.72)/1.93(0.71)^*^	0.97(0.62)/2.91(0.83)^*^	1.25(1.05)/2.91(0.93)^*^	0.69(0.47)/2.22(0.67)^*^	0.399	0.83–1.54	0.5859
Fairness	0.38(0.78)/1.77(1.25)^*^	0.66(0.87)/2.00(1.11)^*^	0.78(0.66)/3.06(0.88)^*^	1.38(1.18)/2.19(1.19)^*^	0.69(0.47)/2.37(0.78)^*^	0.417	0.58–1.35	0.4342

^*^p < 0.05, paired *t*-tests comparing the evaluations for the first and second learning sheets.

^**†**^Slopes are rates of improvement calculated using the fifth learning sheet and the second evaluation scores for the final assessment of improvement. The second evaluation scores for all the other learning sheets were used as linear function factors to plot a quadratic function for each dimension.

**Table 3:** Pharmaceutical students’ overall improvement throughout the five experiments (N = 27)

	*Learning sheets 1* ^*st*^ */ 2* ^*nd*^ *evaluation*						*Overall improvement*	
	**Mean (SD)**					**Slope** ^**†**^	**Multivariate regression**	
**Experiment**	**1**	**2**	**3**	**4**	**5**		**95% CI**	***p***
Dimension								
Clarity	2.44(0.75)/3.56(0.64)^*^	2.85(0.53)/3.00(0.11)^*^	3.41(0.74)/4.00(0.05)^*^	3.41(0.75)/4.00(0.14)^*^	3.00(0.27)/3.00(0.10)^*^	0.212	1.76–5.40	< 0.0001
Accuracy	0.78(0.42)/2.07(0.92)^*^	1.89(0.16)/2.30(0.08)^*^	2.00(1.54)/3.44(0.75)^*^	3.15(0.66)/3.85(0.36)^*^	1.85(0.36)/2.70(0.47)^*^	0.539	0.30–0.82	0.0063
Precision	1.52(0.94)/2.52(1.22)^*^	2.78(0.42)/3.00(0.22)^*^	2.93(0.83)/3.78(0.42)^*^	3.33(0.48)/3.85(0.36)^*^	2.67(0.48)/3.00(0.02)^*^	0.381	0.32–0.85	0.0085
Relevance	2.44(0.93)/2.85(0.66)^*^	2.30(0.47)/2.81(0.40)^*^	2.22(1.25)/3.78(0.42)^*^	2.85(0.66)/3.85(0.36)^*^	1.93(0.92)/2.52(0.51)^*^	0.216	2.15–6.24	< 0.0001
Depth	0.78(0.42)/2.37(0.83)^*^	0.70(0.47)/1.63(0.74)^*^	1.11(1.05)/3.33(1.07)^*^	1.52(0.51)/3.00(0.55)^*^	0.93(0.82)/2.52(0.51)^*^	0.485	0.52–1.03	0.0714
Breadth	1.15(0.36)/2.07(0.92)^*^	0.15(0.36)/0.78(0.70)^*^	0.30(0.47)/0.78(0.70)^*^	1.30(0.47)/3.04(0.93)^*^	0.48(0.75)/2.15(0.66)^*^	0.426	0.60–0.91	0.0045
Logic	0.78(0.70)/2.22(0.80)^*^	1.04(0.81)/2.48(0.51)^*^	1.48(1.25)/3.93(0.27)^*^	3.19(0.74)/3.85(0.36)^*^	1.93(0.92)/2.67(0.48)^*^	0.515	0.32–0.79	0.0027
Significance	0.78(0.42)/2.22(0.80)^*^	1.00(0.55)/2.07(0.73)^*^	1.33(1.24)/2.85(1.43)^*^	1.37(0.49)/3.00(0.55)^*^	0.63(0.74)/2.44(0.51)^*^	0.452	0.98–2.27	0.0628
Fairness	0.93(0.62)/2.37(0.84)^*^	0.81(0.68)/2.00(0.55)^*^	0.81(1.04)/2.85(1.20)^*^	1.56(0.51)/3.04(0.98)^*^	0.63(0.74)/2.52(0.51)^*^	0.422	0.68–1.39	0.8854

^*^p < 0.05, paired *t*-tests comparing the evaluations for the first and second learning sheets.

^**†**^Slopes are rates of improvement calculated using the fifth learning sheet and the second evaluation scores for the final assessment of improvement. The second evaluation scores for all the other learning sheets were used as linear function factors to plot a quadratic function for each dimension.

**Table 4:** Undergraduate medical students’ overall improvement throughout the five experiments (N = 85)

	*Learning sheets 1* ^*st*^ */ 2* ^*nd*^ *evaluation*						*Overall improvement*	
	**Mean (SD)**					**Slope** ^**†**^	**Multivariate regression**	
**Experiment**	**1**	**2**	**3**	**4**	**5**		**95% CI**	***p***
Dimension								
Clarity	2.21(1.03)/3.37(0.83)^*^	2.23(0.86)/2.81(0.39)^*^	3.22(1.02)/3.50(0.85)^*^	3.59(0.68)/4.00(0.05)^*^	2.71(0.55)/3.00(0.12)^*^	0.277	1.43–2.70	< 0.0001
Accuracy	0.84(0.80)/2.29(0.78)^*^	1.40(1.00)/2.41(0.68)^*^	2.22(1.18)/3.15(1.10)^*^	3.09(0.78)/3.51(0.50)^*^	2.56(0.59)/2.89(0.31)^*^	0.520	0.46–0.80	0.0003
Precision	1.78(0.89)/2.88(0.73)^*^	1.87(0.73)/2.56(0.58)^*^	2.33(1.03)/3.42(0.90)^*^	3.50(0.85)/3.94(0.24)^*^	2.66(0.57)/2.94(0.24)^*^	0.370	0.57–1.13	0.1987
Relevance	1.29(0.63)/2.49(0.81)^*^	1.85(0.79)/2.35(0.72)^*^	2.87(0.95)/3.45(0.90)^*^	3.05(0.94)/3.72(0.45)^*^	2.53(0.66)/2.84(0.37)^*^	0.447	0.70–1.20	0.5249
Depth	0.94(0.74)/2.41(0.96)^*^	1.42(1.10)/1.94(1.75)^*^	0.86(0.73)/2.89(1.21)^*^	1.04(0.98)/2.99(1.17)^*^	1.52(0.81)/2.71(0.55)^*^	0.459	0.58–0.93	0.0092
Breadth	0.88(0.69)/2.16(1.19)^*^	0.95(1.02)/1.36(0.99)^*^	0.48(0.69)/2.47(1.27)^*^	0.81(0.68)/2.78(1.08)^*^	1.02(0.61)/1.95(0.82)^*^	0.356	1.05–1.47	0.0100
Logic	0.80(0.75)/2.41(0.87)^*^	1.50(0.94)/2.29(0.85)^*^	2.05(1.31)/3.05(1.08)^*^	3.05(0.94)/3.55(0.45)^*^	2.64(0.59)/2.89(0.31)^*^	0.544	0.70–0.97	0.0190
Significance	1.05(0.88)/2.24(0.89)^*^	1.35(1.04)/2.14(0.91)^*^	1.11(1.11)/2.72(1.17)^*^	0.90(0.70)/2.49(1.10)^*^	1.13(0.68)/2.51(0.73)^*^	0.327	1.06–2.09	0.0225
Fairness	1.06(0.83)/2.56(0.82)^*^	1.24(0.85)/2.14(0.81)^*^	1.36(1.05)/3.05(1.19)^*^	0.99(0.85)/2.45(2.25)^*^	1.17(0.71)/2.44(0.81)^*^	0.307	0.75–1.26	0.8295

^*^p < 0.05, paired *t*-tests comparing the evaluations for the first and second learning sheets.

^**†**^Slopes are rates of improvement calculated using the fifth learning sheet and the second evaluation scores for the final assessment of improvement. The second evaluation scores for all the other learning sheets were used as linear function factors to plot a quadratic function for each dimension.

### Trajectory tracking of the overall, significant, and reciprocal dimensions (Aim 2 and Aim 3)

**Fig. 2:**
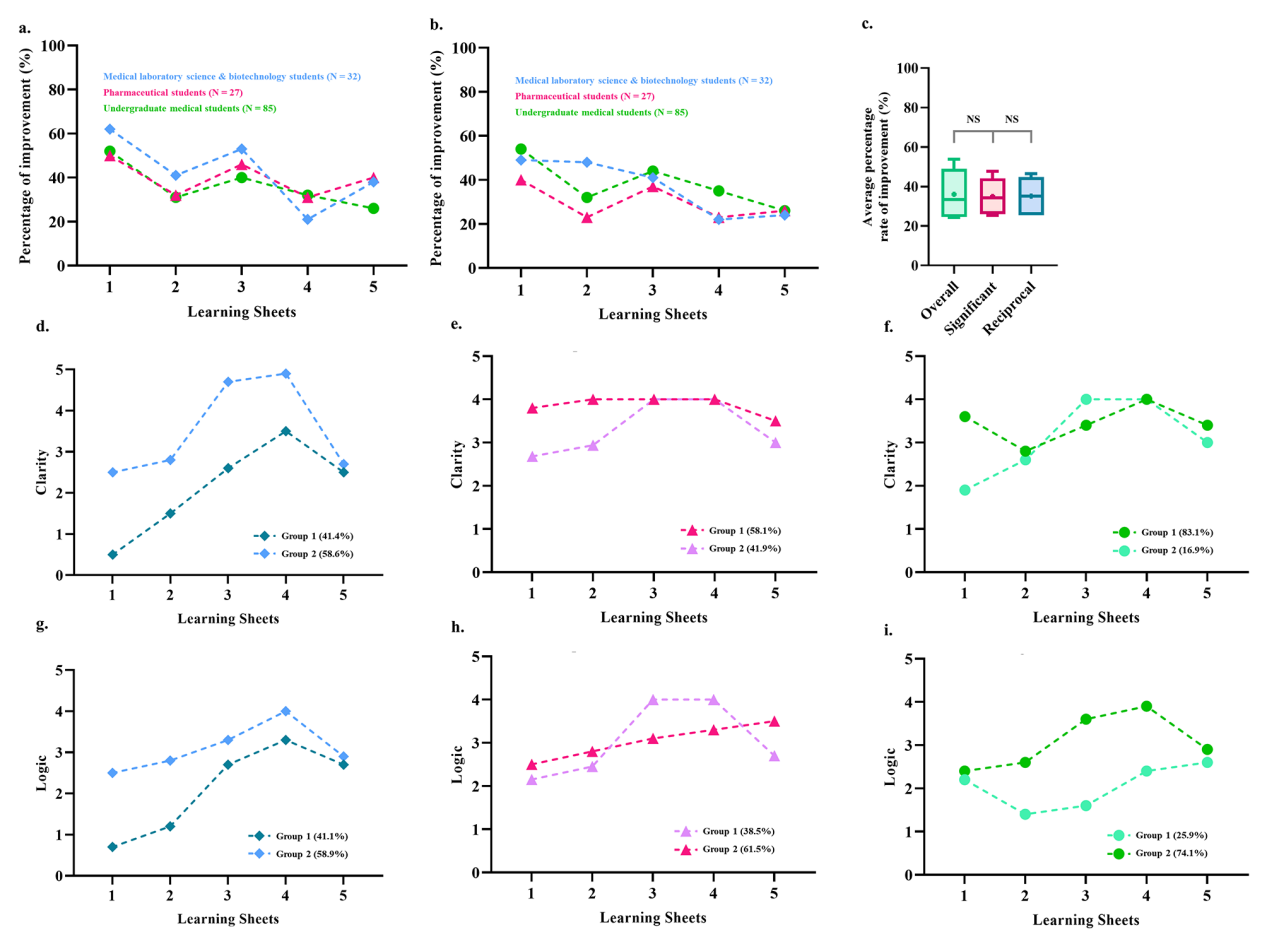
**Overall improvement comparison between the students of three majors using a trajectory-tracking analysis approach**. (**a**) The mean evaluation scores from the second evaluation minus those from the first evaluation for the nine dimensions were considered an improvement. They were converted to percentages to compare them to the performance in the first evaluation. (**b**) The mean evaluation scores from the second evaluation minus those from the first evaluation for the significant dimensions (within the students of each major, Tables 2–4) were considered to represent improvement and were converted to percentages to compare them to the performance in the first evaluation. (**c**) Comparison of the average percentage improvement among all nine dimensions, the significant dimensions, and the reciprocal dimensions (i.e., clarity and logic). (**d**) Trajectory analysis to assess the progress of the two subgroups of medical laboratory science and biotechnology students in the clarity dimension. (**e**) Trajectory analysis to assess the progress of the two subgroups of pharmaceutical students in the clarity dimension. (**f**) Trajectory analysis to assess the progress of the two subgroups of undergraduate medical students in the clarity dimension. (**g**) Trajectory analysis to identify the progress of the two subgroups of medical laboratory science and biotechnology students in the logic dimension. (**h**) Trajectory analysis to assess the progress of the two subgroups of pharmaceutical students in the logic dimension. (**i**) Trajectory analysis to assess the progress of the two subgroups of undergraduate medical students in the logic dimension

Figure 2a illustrates the overall improvement of students across the three majors in all nine dimensions, as assessed via trajectory analysis. The trajectory-tracking algorithm revealed that the significant dimensions for each group were as follows: MSB students—clarity, relevance, and logic; PS students—clarity, accuracy, precision, relevance, breadth, and logic; and MU students—clarity, accuracy, depth, breadth, logic, and significance (Tables 2, 3 and 4; Fig. 2b). The comparison of each group’s average percentage of improvement between the nine dimensions, the significant dimensions, and the reciprocal dimensions (clarity and logic) is summarized in Fig. 2c. Figure 2d–i depicts the students’ improvement in clarity and logic within the different majors using group-based trajectory modeling.

## Discussion

### Empirical contributions

The Han Chinese educational system relies on the passive transmission of knowledge, as evidenced by the years of preparation by students’ through paper-based exams. By adopting this approach during teaching and learning, students do not develop a critical thinking mindset. Our experience has shown that when we encounter first-year students who have just graduated from high school, their previous education failed to develop critical thinking skills. Many foreign and Western teachers have the same experience when they encounter Asian students studying abroad for the first time. Thus, this research aims to provide clinical teachers with guidance on reducing the blind spots that students face when introduced to critical thinking. Moreover, this research aims to provide teachers with a simple teaching model and structure to guide students with less stable foundations in critical thinking. For the teaching structure and process, please refer to the procedure paragraph in the methods section and the teaching flow chart in Fig. 1. Furthermore, the scoring system shown in the assessment development paragraph in the methods, as well as the scoring rubric is presented in Table S1.

To our knowledge, this is the first study that uses the Socratic method and the universal intellectual standards to assess and improve critical thinking skills in biochemistry laboratory courses across different healthcare majors. We also used a novel design for teaching critical thinking, with multi-timepoint assessments and trajectory-tracking analysis to observe the students’ process and the improvement intheir critical thinking. This Socratic method, combined with critical thinking-based learning sheets, significantly improved the students’ critical thinking in all nine dimensions of the universal intellectual standards, according to the first and second evaluations conducted in each of the five sessions. Another unique contribution of this study is that it analyzed the progression results at multiple time points in the critical thinking performance of students across different majors. According to the results of comparing the average percentage improvement between all nine dimensions, the significant and reciprocal dimensions (i.e., clarity and logic) do not significantly differ from each other statistically speaking. By reducing the nine intellectual dimensions scoring system, medical educators can focus more on establishing clarity and logic skills in students. In sum, our most important finding was the identification of the clarity and logic dimensions as key elements that facilitate the development of critical thinking skills via the Socratic method in students across three different healthcare majors.

### The trajectories of outcomes for students of medical science and biotechnology majors

Understanding what we learn has been identified as the starting point in the professional-development journey [2]. In principle, if thinking and decision making can be taught, educational intervention is possible. Nevertheless, for a science class like biochemistry, abductive reasoning requires a deep understanding of knowledge, and thinking must be inspired through stimulation.

In this study, the evaluation scores for MSB students did not improve significantly in almost any dimension at the beginning of the course. At first, most students felt uncomfortable with criticizing others, disagreeing with others, or challenging teacher’s knowledge and authority when they spoke their minds. Other MSB students believed that their ability to find answers and make decisions was inadequate and expected the teacher to provide the correct answers. However, preclinical medical technologists must gradually develop their critical thinking skills. Thus, the teacher provided critical thinking cues during the class and monitored the group discussions.

On the other hand, teachers must encourage these types of students, enabling them to accomplish simpler learning goals by providing them with easier-to-attempt clues. The joy of discovering answers on their own rather than the frustration of not achieving high goals should be encouraged. This coaching process improved the MSB students’ willingness to think and explore, leading to greater relevance and breadth of coverage.

The teacher used generation, conceptualization, optimization, and implementation [33] with the Socratic method to stimulate critical thinking in a four-step cycle in the five experiments. When the spontaneous discussion started in the generation phase, they tried to clarify their knowledge of the theme and identify the problem from the learning sheet. The following step was to conceptualize the problem, and the students drafted all of the possibilities and problems. Teacher frequently asked the students, ‘*What are other possible reasons?*’ Finally, the teacher provided feedback to help the MSB students reach a proper solution and implement it. The teacher would also ask the students leading questions like ‘*What relevant theories can be confirmed more precisely?*’ These guiding processes sharpened their logic and helped them better understand what they had learned. In sum, the benefits of this process included an enhanced ability to think logically, clarification of questions and knowledge gaps, and improvements in the thought process about the theme discussed.

### The steady improvement of critical thinking in the students of pharmaceutical science

Currently, pharmacists are seeing their roles and responsibilities shift to becoming patient counselors and educators on the rational use of medicine. Pharmacists are trained to focus on patient-centered care and resolve current and potential drug-related problems [83, 84]. Critical thinking, clinical reasoning, and decision-making skills are needed to solve these problems. Nowadays, pharmacists are not just responsible for carrying out doctor’s orders, while there are always alternative treatment options available for them to recommend. Teacher therefore repeatedly emphasized the link between critical thinking and pharmacist practice and encouraged students to ask questions and find out the best alternative through Socratic method in the classroom.

During class, the PS students were required to exert considerable mental effort to conduct an inquiry to solve the learning sheet questions. Instead of providing students with clues or information to help them solve the problems, the teacher guided the PS students on how to seek the information they needed for themselves. The question for the PS students was be ‘*What are the possibly executable strategies?*’ The teacher also joined the students in discussion, using the Socratic method to stimulate critical thinking and draw out ideas and underlying suppositions. In high-quality cooperative argumentative dialogue, teacher should not direct or refer learning, nor should they ask students for the correct answers as in a traditional classroom. The hints that teacher would provide were more like ‘*The narrative explanation can be more precise.*’ Thus, asking high-quality questions and providing feedback also challenges the instructors’ teaching experience.

The PS students were guided not only toward the development of critical thinking skills but also toward solving problems using evidence-based knowledge and decision-making skills. The Socratic method process meets the student where they are on the educational spectrum and encourages and helps them advance. Using this method, the PS students engaged in student-to-student interaction to build knowledge as a group and individually. The course of five experiments conducted via the learning sheets improved many aspects of the students’ critical thinking, including their clarity, relevance, breadth, and logic. In sum, the abilities that they developed in the course should help them focus more on the possible outcomes of pharmacotherapy, medication surveillance, and proper communication and therefore improve the quality of their professional future.

### The advanced construction of critical thinking skills in undergraduate medical students

In medical education, “*better thinking and learning skills grounded in understanding*” are recommended for future doctors [2]. Practicing medicine requires an ability to address current and future diseases using new diagnostic and therapeutic methods [10]. Therefore, problem solving is not the only core medical skill; the ability to deal with complex, insoluble health issues is also required [83]. In this domain, critical thinking skills have proven essential in tackling difficult, complex, interdisciplinary health problems [10].

In our study, the MU students began with high-performance scores in almost all dimensions. As a result, teachers needed to create a more challenging and thought-provoking learning environment to encourage them to think more broadly and deeply. Thus, the teacher would give students advice like ‘*Searching for more relevant information can increase the breadth of knowledge*’ and ‘*If the result is true, what is the relevant theory?*’ Most MU students were faster than other majors at defining and constructing critical thinking. However, another phenomenon often observed in the classroom was that the MU students were more reluctant to express their reasoning than the students of other majors. In other words, MU students were afraid to speak openly about their reasoning and thinking, probably due to the excessive pursuit of the correct answer. In sum, the course of five experiments conducted via the learning sheets enhanced abilities of clarity, accuracy, depth, breadth, logic, and significance in MU students.

Apart from providing structure for their critical thinking, as was done with the other preclinical students, the teacher guided the MU students to use advanced critical thinking skills by regularly analyze their thinking processes, reflecting on the decision-making and thinking process [84]. Researchers have suggested that reflective practice is key to successful medical professionalism [85] and humanism [86, 87]; but more importantly, it may help medical professionals develop better physician–patient relationships [88]. Therefore, to advance the critical thinking experience of the MU students, teacher should encourage them to gather ideas, analyze, evaluate, and synthesize information. The teacher guided them to reflect on their plan and solve the questions on the learning sheets using their thoughts and words. These reflective practices could involve various biases in the thinking process and outcome, such as the base-rate fallacy, bias blind spot, or choice-supportive bias. The Socratic debate is a common way to model a complex thinking situation and may help teachers inspire students to become critical thinkers. MU students improved their abilities in the clarity, accuracy, depth, breadth, logic, and significance dimensions in the five experiments. This kind of training in thinking should help preclinical students constantly challenge and critically appraise evidence within their context, as well as their patients’ and their own belief and value systems.

### Limitations

This study provides a model for developing a specific learning environment like a biochemistry laboratory class into one that will help students develop their critical thinking skills through inquiry. Our results have shown this method to be feasible and effective. However, there were a few limitations to this study. First, although it included students from three different majors, there was no interdisciplinary collaboration that would have simulated collaborations and communication among other healthcare professionals from different fields, as occurs in clinical practice. Introducing such collaboration may have produced more exciting and comprehensive ideas for solving the problems. Training in these professions is specialized to a considerable extent, so inter-professional collaboration should improve therapeutic outcomes and optimize patient care. Second, the original scoring system was time-consuming. However, one of our study objectives was to modify and reduce the nine intellectual dimensions scoring system into the clarity and logic dimensions. Based on the analysis in the current study, the clarity and logic dimensions were sufficient for monitoring the growth of students’ critical thinking.

## Conclusion

The present curriculum innovation aimed to teach critical thinking skills to preclinical students in various medical majors using a Socratic questioning learning model instead of a cookbook approach to learning in laboratory courses. The development of problem-solving and critical thinking skills, in addition to process-related skills, in biochemistry laboratory courses supplements traditional curriculum in a helpful way. The curriculum innovation that we described and proposed may represent an incremental step forward for the discipline; it is a novel educational approach for promoting critical thinking skills, fostering an appreciation of the affective domain, and enabling reflective practice by using small-group processing skill instruction and one-on-one Socratic questioning. The current study results are based on training critical thinking skills that should enable students to engage in the “reflection-on-action” process, which might provide an additional bridge between basic medical knowledge and clinical practice. More importantly, reconstructive mental reviews may indirectly shape preclinical students’ future actions in the challenging healthcare industry characterized by uncertainty and novel circumstances.

## Electronic supplementary material

Below is the link to the electronic supplementary material.

Supplementary Material 1

## Data Availability

Due to conditions on participant consent and other ethical restrictions, the datasets used and analysed in the current study are not publicly available. If you have any database data requirements, please contact the corresponding author of this study.
