# Supplementary material for: Thinking more wisely: using the Socratic method to develop critical thinking skills amongst healthcare students
Source: BMC Med Educ. 2023 Mar 20;23:173. doi: 10.1186/s12909-023-04134-2 (PMC10026783; doi:10.1186/s12909-023-04134-2)
Supplement: Supplementary file 1 — Supplementary Material 1 [file 12909_2023_4134_MOESM1_ESM.docx]

**Supplementary Information**

| **Table S1 Socratic questions and students’ answers.** | |
| --- | --- |
| Inquiry questions | Student answers |
| If the viral load is too low, or PCR is performed erroneously, the test result will be negative. Can such patients be clinically diagnosed as not infected? | **Principle:** The presence of the band indicating electrophoresis in the PCR screening (test) means the test result is positive, the virus is present in the specimen, and the patient is infected by the virus. On the other hand, if the band does not appear, the test is negative; in other words, there is no virus detected in the clinical specimen, and the patient is not infected by the virus.  **Clinical medicine:** Various errors that can be caused by multiple factors have not been excluded. Different viruses need different screening times. If the PCR test is negative and the negative result is confirmed, the patient should be regarded as not infected. |
| Are there any other techniques that are more sensitive than a PCR test? | The PCR test is negative because the viral load is below the minimum detection threshold; this does not mean that the screened individual is not infected with the virus. In addition, inappropriate sampling may also result in a negative test. In clinical practice, multiple screenings and different techniques (methods) are often used to test clinical specimens to confirm a negative result, with reference to the clinical manifestations and characteristics. |

| **Table S2** **Critical thinking rubric, based on the concept of intellectual standards elaborated by Drs Linda Elder and Richard Paul.** | | |
| --- | --- | --- |
| Criteria | Scale | |
|  | 0 | 1 |
| Clarity | The description is considered out of scope and incomprehensible. | The description is considered highly relevant and comprehensible. |
| Accuracy | The description does not comply with the existing scientific theories. | The description complies with the known scientific theories. |
| Precision | The description and wording are not academic. | The description and wording are at the academic level. |
| Relevance | The description is irrelevant. | The description is highly relevant. |
| Depth | The description contains basic information. | The description contains arguments based on insightful aspects. |
| Breadth | The description contains limited arguments. | The description contains fully fledged arguments. |
| Logic | The description is illogical and incoherent. | The description is logical and coherent. |
| Significance | The description does not include critical thoughts. | The description includes critical thoughts. |
| Fairness | The description includes subjective arguments. | The description includes objective arguments. |
